# Absence of convincing evidence of *Coxiella burnetii* infection in Chile: a cross-sectional serosurvey among healthy adults in four different regions

**DOI:** 10.1186/s12879-016-1880-9

**Published:** 2016-10-06

**Authors:** Thomas Weitzel, Javier López, Gerardo Acosta-Jamett, Sophie Edouard, Philippe Parola, Katia Abarca

**Affiliations:** 1 Laboratorio Clínico, Clínica Alemana de Santiago, Facultad de Medicina Clínica Alemana, Universidad del Desarrollo, Santiago, Chile; 2 Hospital Veterinario Puente Alto, Santiago, Chile; 3 Instituto de Medicina Preventiva Veterinaria, Facultad de Ciencias Veterinarias, Universidad Austral de Chile, Valdivia, Chile; 4 Unité de Recherche sur les Maladies Infectieuses et Tropicales Emergentes, IHU Méditerranée-Infection, Aix-Marseille Université, Faculté de Médecine, Marseille, France; 5 Departamento de Enfermedades Infecciosas e Inmunología Pediátrica, Escuela de Medicina, Pontificia Universidad Católica de Chile, Santiago, Chile

**Keywords:** Q fever, *Coxiella burnetii*, Epidemiology, Seroprevalence, Zoonoses, South America

## Abstract

**Background:**

*Coxiella burnetii* is an important zoonotic pathogen of global distribution. Still, in most parts of South America including Chile, systematic epidemiological data are lacking. The presented study aims to determine the seroprevalence of *Coxiella burnetii* antibodies in healthy adults of four different regions in Chile.

**Methods:**

A cross-sectional study was performed, which included healthy adults living in rural and urban areas of four cities located in different regions in northern, central, and southern Chile. In urban sectors, households were chosen by double stratified random sampling, while in rural areas convenience sampling was performed. Serum specimens were taken and screened for the presence of IgG antibodies against *C. burnetii* phase II antigen using a commercial ELISA kit. Positive and indeterminate results were confirmed by a reference laboratory using indirect immunofluorescence assay (IFA).

**Results:**

A total of 1112 individuals were included. Of those, 8 were positive by ELISA, but only one sample was confirmed using IFA. Statistical analysis for population freedom from disease revealed a high probability that *C. burnetii* was absent in our study population.

**Conclusion:**

Our work provides the first epidemiological data on human Q fever in Chile indicating either a very low endemicity or the absence of this pathogen in the studied areas.

**Electronic supplementary material:**

The online version of this article (doi:10.1186/s12879-016-1880-9) contains supplementary material, which is available to authorized users.

## Background

*Coxiella burnetii* is a Gram-negative intracellular coccobacillus that belongs to the order of Legionellales. This obligatory intracellular zoonotic pathogen infects various domestic and wild mammals, birds, and arthropods such as ticks. The most important reservoirs for human infections are domestic ungulates such as cattle, sheep, and goats, although pets can also become infected [1]. Transmission occurs by inhaling or ingesting spores, ingestion of unpasteurized milk or dairy products, through sexual contact, and by vectors such as ticks. While infections of animals often stay asymptomatic, human infections might present as acute or chronic Q fever. Often, the symptoms are discrete and nonspecific (“query fever”) [2]. It is assumed that *C. burnetii* is a global zoonotic disease, which is endemic or occurs sporadically in all countries with the exception of New Zealand and French Polynesia [3]. In South America only few epidemiological data have been published and systematic studies are lacking [3, 4]. In Chile, zoonotic Q fever has been detected and the infection is listed among the notifiable animal diseases [5]. However, systematic studies and scientific publications on the epidemiology of zoonotic and human Q fever in Chile are scarce. The aim of this study was to determine the seroprevalence of anti-*Coxiella burnetii* antibodies in healthy inhabitants of four different Chilean regions and to analyze possible geographical differences.

## Methods

### Samples

**Fig. 1:**
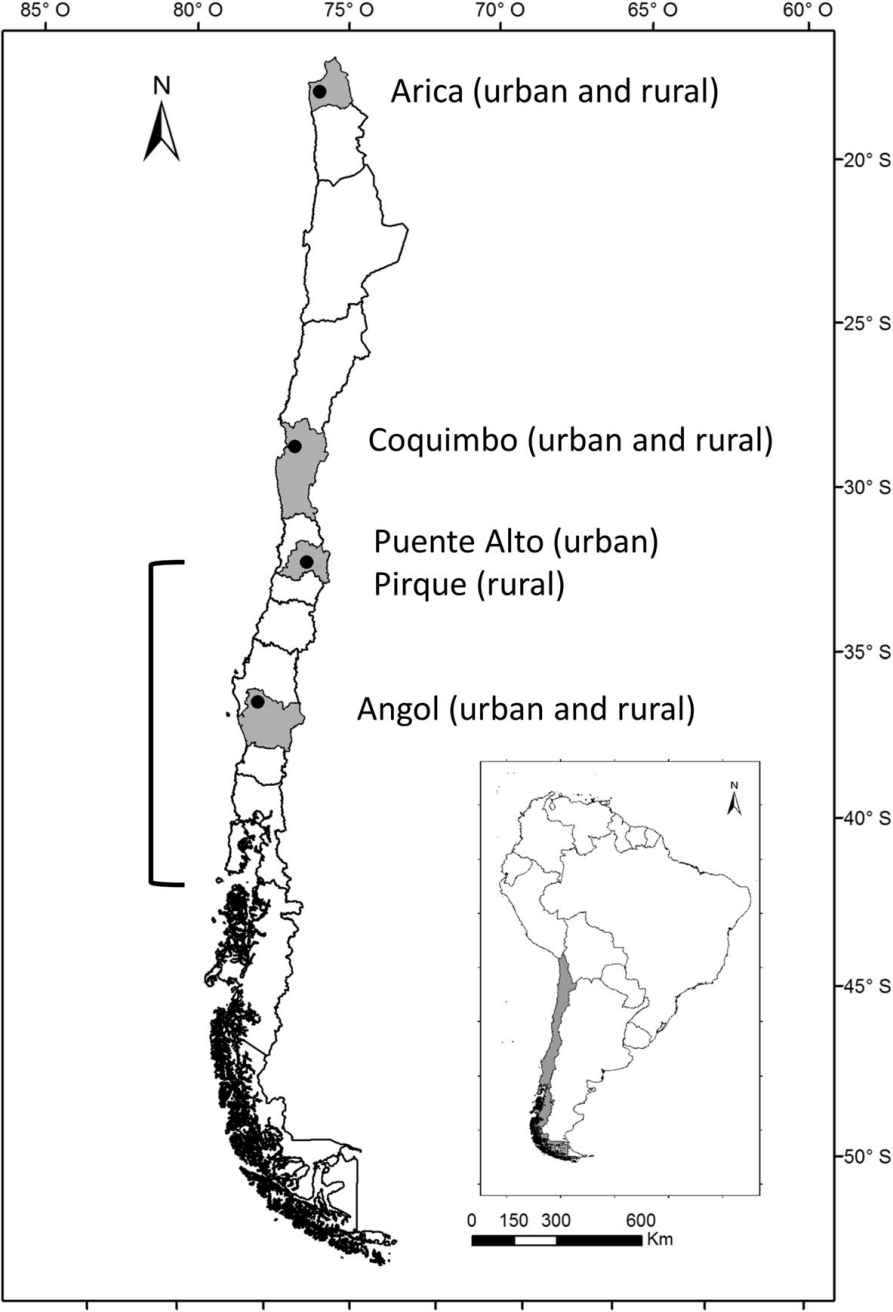
Study sites and reported zoonotic *C. burnetii* infections in Chile. Grey shading and dots represent the study regions and cities, respectively. The bracket indicates the regions where zoonotic infections have been reported previously [29, 31]

The samples of this work derived from an ongoing field project studying various vector-borne zoonotic infections in humans, canines, and vectors in Chile. It was conducted in four regions (Fig. 1) and included the following areas: 1) the city of Arica and surrounding rural areas of the Arica y Parinacota Region (18°28′S,70°18′W), which is located in the far north of Chile, with dry climate, extremely arid landscape and little vegetation, and is home to 180,879 inhabitants; 2) the city of Coquimbo (29°57′S,71°20′W) and surrounding rural areas in the Coquimbo Region, with semi-arid climate and a population of 203,036 inhabitants; 3) the municipality of Puente Alto in the Metropolitan Region (33°37′S,70°34′W) with 492,915 inhabitants, and a the nearby rural municipality of Pirque, with Mediterranean climate and extended dry season; and 4) the city of Angol and rural areas in the Araucanía Region (37°48′S,72°43′W) located in southern Chile, with a transitional climate from humid mild Mediterranean to markedly rainy and 42,000 inhabitants. The study was performed between September 2010 and January 2011 in the Arica y Parinacota and Metropolitan regions and between October 2011 and February 2012 in the Coquimbo and Araucanía regions. The numbers of inhabitants were derived from the most recent census figures [6]. A sample size of 114 people per each area was calculated using an estimated prevalence of 5 %, confidence interval of 95 % and an error of 4 %. In the absence of data from Chile, this estimation based on studies from Brazil and Uruguay (see Additional file 1: Table S1). In urban areas, a double stratified random sampling per building block and household was carried out while in rural areas a convenience sampling was performed until the number of pre-established households was completed. Individuals were informed about the study and after written consent was obtained, blood was drawn from one or two adult members of each household. Specimens were centrifuged on the same day and serum was separated, aliquoted, and kept at −20 °C until further analysis.

### Serological screening by ELISA

Samples were transported at −20 °C to Santiago, Chile, where ELISA testing was performed. Sera were screened using SERION ELISA classic *Coxiella burnetii* Phase II IgG (Virion/Serion, Würzburg, Germany) according to the manufacturer’s instructions. Samples were diluted 1:500 with dilution buffer; then 100 μL of diluted sera were added to each well of 96-well plates coated with *C. burnetii* phase II antigen. Each run included the respective cut-off and negative controls provided by the manufacturer. Plates were processed and analyzed according to the manufacturer’s instructions with a fully automated 4-plate ELISA processing system (Immunomat™, Virion/Serion). The assay provides quantitative results by using one-point calibration of a standard curve with values <20 U/mL considered negative and >30 U/mL positive. In a previous evaluation, the assay showed a sensitivity and specificity of 89 and 97 %, respectively [7].

### Serological confirmation by immunofluorescence

Aliquots of equivocal or positive samples by ELISA were sent on dry ice to a reference laboratory (Unité des Rickettsies, Marseille, France), where confirmatory tests were performed. Titers of IgG, IgM, and IgA antibodies were determined using an indirect immunofluorescence assay (IFA) with highly purified antigens generated in-house [8]. Specimens were diluted at ratios of 1:50 and 1:100 and screened for total immunoglobulin. If the serological screening for Q fever was positive with either dilution, final titers for IgG, IgA, and IgM were determined for both anti–phase I and anti–phase II antibodies. IFA-titers of phase II IgG ≥1:200 and phase II IgM ≥50 indicate acute Q fever, whereas a phase I IgG titer ≥ 1:800 suggests a persistent infection in patients with cardiovascular predisposition [9]. The presence of Phase I or II IgG titers between 1:100 and 1:400 in the absence of IgM and IgA are related to past infection [10]. For our study, seropositivity was defined when antibody titers against any of the phases were 1:100 or above.

### Statistical analyses

Sample size calculation was performed by Epi Info™ 7. The probability that *C. burnetii* was absent (freedom from disease) was estimated as described by Cameron and Baldock [11] using EpiTools (http://epitools.ausvet.com.au/content.php?page=FreeCalc1) under the following assumptions: prevalence 5 %, ELISA sensitivity 89 % and specificity 97 %, IFA sensitivity 86 % and specificity 93 % [10], and error margins of 5 %.

### Ethical approval

The study protocol was reviewed and approved by the Comité Ético Científico of the Faculty of Medicine, Pontificia Universidad Católica de Chile in Santiago, Chile, and by the respective health authorities of the four study regions.

## Result

A total of 1112 individuals were included and tested. Of those, 555 (49.9 %) lived in an urban and 557 in a rural environment, 721 (64.8 %) were female, 71 (6.4 %) were full-time farmers, and the median age was 46 years (range: 18–92 years). Screening by *C. burnetii* Phase II IgG ELISA revealed eight positive and four indeterminate results, but only one sample was confirmed by the serological reference tests (Table 1). The positive specimen had low positive IgG titers (1:100) against *C. burnetii* phase I and II antigens probably related to a past infection. To examine possible cross-reactivity, this sample was tested with a panel of serological tests including *Rickettsia conorii*, *R. typhi*, *R. felis*, *Francisella tularensis*, *Anaplasma phagocytophillum*, *Bartonella henselae*, and *B. quintana*, which were entirely negative. The specimen derived from an 86 year-old man living and working in a rural area of Angol in southern Chile.

**Table 1:** Antibodies against *C. burnetii* antigens in 1112 samples from four regions in Chile

Study site		ELISA (screening)				IFA (confirmation)		
(Region/city)	Setting	N	Neg.	Indet.	Pos.	n	Neg.	Pos.
Arica y Parinacota/Arica	Urban	121	118	2	1	3	3	0
	Rural	130	129	0	1	1	1	0
Coquimbo/Coquimbo	Urban	148	146	1	1	2	2	0
	Rural	141	141	0	0	0	0	0
Metropolitan/Puente Alto	Urban	146	145	1	0	1	1	0
	Rural	144	143	0	1	1	1	0
Araucanía/Angol	Urban	140	139	0	1	1	1	0
	Rural	142	139	0	3	3	2	1
Total	All	1112	1100	4	8	12	11	1

*ELISA*, enzyme- linked immunosorbent assay, *IFA*, indirect immunofluorescence assay

The estimated values of probability of freedom from *C. burnetii* seroreactivity for the entire population and populations from different regions were >99 and >97 %, respectively, using the ELISA results, and >99 and >99 %, respectively, for the IFA results.

## Discussion

The main diagnostic tool for human *C. burnetii* infections is serology [3, 12]. ELISA might be used as a screening test, but indirect immunofluorescence assays (IFA) is considered the serological gold standard due to its high sensitivity and specificity [1]. Prevalence studies are mainly based on the detection of antibodies against phase II antigen, which after infection remains detectable for years or for life [2]. For our study, we chose a two-step approach, ELISA screening followed by IFA confirmation, which has been used in other epidemiological surveys [13, 14]. This approach increases specificity, since ELISA is sensitive but prone to cross-react with other intracellular bacteria such as *Legionella* or *Bartonella* [12]. IFA, as a quantitative test, helps to exclude false-positive ELISA results, since the responsible antibodies usually reach low titers [13]. Therefore, some authors have recommended increasing IFA cut-off levels [15, 16]. In our study IgG IFA titers ≥1:100 were considered positive. This cut-off has been evaluated for clinical use [10] and has been used in various epidemiological studies [14, 17, 18]. Still, there is no international consensus on the IFA cut-off value for seroprevalence studies, and other studies use lower or higher levels [13, 19–21], which is a major constraint in the comparison of epidemiological data of this pathogen [22].

*Coxiella burnetii* is considered to occur worldwide (except New Zealand and French Polynesia); however, its incidence rates vary considerably from country to country [23]. Because of its clinical polymorphism, the diagnosis of Q fever is challenging [1]. With an increasing awareness and diagnostic advances, the infection has emerged in various regions including the tropics [3]. In France, on the other hand, the knowledge of Q fever and prevention measures have led to a decrease of Q fever endocarditis cases over the last years [24]. In many other countries, especially within the non-industrialized world, there is a lack of awareness, diagnostic tools, and reference laboratories, and the epidemiological relevance of Q fever remains obscure [12, 25]. This is the case for wide parts of South America, where few epidemiological studies have been published and there is an apparent deficit of comprehensive and up-to-date data. A recent critical review of the global prevalence of *C. burnetii* in domestic ruminants, for example, summarized data from all five continents, but did not include a single study from South America [26]. Still, if older and non-English language publications are included, there are reports of serological evidence in domestic animals from Argentina, Brazil, Colombia, Uruguay, and Venezuela (see Additional file 1: Table S1). Another study detected genetic material of *C. burnetii* in cow milk in Ecuador and a recent report found *C. burnetii* in ticks in Argentina (for references see Additional file 1: Table S1). More conclusive data on the existence and reservoir of *C. burnetii* are only available in French Guiana, where Q fever is a frequent cause of pneumonia and seems to be linked to sylvatic animals including three-toed sloths [27, 28]. Human *Coxiella* infections have been diagnosed or suspected in patients from Argentina, Brazil, Colombia, Ecuador, French Guiana, Peru, Uruguay, and Venezuela, but most of these reports represent single case reports (see Additional file 1: Table S1).

The zoonotic situation of Q fever in Chile is uncertain since epidemiological studies have not been published. The World Organisation for Animal Health (OIE) lists Chile among the countries reporting Q fever in animals, which is based on serological results in 5 of 98 asymptomatic sheep in the Los Lagos region in southern Chile in 2006 [29]; the last positive cases were reported by national authorities in 2007 [30]. According to an expert meeting held by the Chilean Society of Infectious Diseases in 2001, zoonotic infections have occurred in the Metropolitan Region and different regions in southern Chile (Fig. 1) [31]. In recent years, two unconfirmed clusters of Q fever were reported in alpacas, which were imported from northern Chile into China [32, 33]. Epidemiological studies or clinical cases of Q fever in humans in Chile have not been published yet, although there is anecdotic information of a cluster of human cases after occupational exposure to imported sheep, which appeared in a quarantine station 12 km east of Santiago in 1998 [34]. Until now, it was uncertain, if this paucity of cases was related to the absence of the pathogen or to underreporting, since routine serological tests for human samples are not available in Chile.

Chile has unique geographical characteristics; bordered by the Pacific Ocean and the Andean mountain belt, it has an island-like character and epidemiological patterns of infectious diseases might differ substantially from neighboring countries. On the other hand, Chile has a length of over 4.200 km including various biogeoclimatic zones, which is a challenge for epidemiological surveys. Although our study only examined samples from four of the 15 Chilean regions, the study sites were located over a distance of more than 2.150 km and included northern, central and southern parts of the country (Fig. 1). The study provides the first epidemiological data on human exposure and infection with *C. burnetii*. Among the more than 1000 samples, only one specimen had IFA confirmed *C. burnetii* antibodies above our threshold. It is important to consider that in situations of very low prevalence (≈0.1 %) the positive predictive value of even highly specific tests is considerably low. Furthermore, it is unknown if the 86-year old participant with the positive serology had previously lived outside Chile. To examine the likelihood that *C. burnetii* was absent in our study population, we used a “proof of freedom from disease” model for imperfect tests [11], which indicated a high probability that *C. burnetii* infection was absent in the studies regions in Chile. This conclusion applied to the ELISA and the IFA results and was independent of the study population (entire study population vs.individual regions).

Limitations of our study design were that sites were not systematically chosen (e.g., to represent different biogeoclimatic zones) and various southern regions, where sheep and cattle farming are important agricultural sectors and where zoonotic infections have occurred [31], were not included. Furthermore, information on risk factors for Q fever such as animal exposure and consumption of unpasteurized milk or dairy products were not available. Our cross-sectional study design also included low-risk urban areas and, since sampling was performed during daytime household visits, females, who might be less exposed to *C. burnetii* [13, 18, 19], were overrepresented.

The knowledge of the local Q fever epidemiology is necessary for the management of possible clinical cases. If there is a high prevalence, serology has to be interpreted with caution, since positive results might be caused by past exposure or infection. On the other hand, if Q fever is very rare or absent, routine diagnostic testing for Q fever as recommended for patients with certain clinical presentations such as unexplained heart valve disease [3], might be dispensable. In these low endemic regions, most positive results might be related to cross-reactions, which can be caused by *Legionella* spp. and *Bartonella* spp. [12]. Especially the latter could be of relevance in Chile, since prevalence rates of more than 10 % have been reported [35]. In such settings, seroepidemiological surveys assays based only on ELISA will overestimate the true prevalence. In our study, for example, 11 of 12 samples with positive or indeterminate ELISA results were not confirmed by IFA testing.

## Conclusions

Our study provides first epidemiological data on human *C. burnetii* infection in Chile indicating that this pathogen might be of very low endemicity or non-endemic in the studied areas. This information is a step forward in understanding the epidemiology of Q fever in this part of South America. Still, to confirm the very low rate or absence of this zoonotic infection in Chile, further systematic serological surveys including larger numbers of participants and/or molecular studies in animals, humans, and potential vectors should be performed.

## Additional file

Additional file 1: Table S1. OIE status and published data on *Coxiella burnetii* infections in animals and humans in South America. (PDF 107 kb)
