# Supplementary material for: Absence of convincing evidence of Coxiella burnetii infection in Chile: a cross-sectional serosurvey among healthy adults in four different regions
Source: BMC Infect Dis. 2016 Oct 6;16:541. doi: 10.1186/s12879-016-1880-9 (PMC5054538; doi:10.1186/s12879-016-1880-9)
Supplement: Additional file 1: Table S1. — OIE status and published data on Coxiella burnetii infections in animals and humans in South America. (PDF 107 kb) [file 12879_2016_1880_MOESM1_ESM.pdf]

Additional File

Table S1. OIE status and published data on *Coxiella burnetii* infections in animals and humans in South America

Country	OIE status[a]	Animal infections (diagnosis)	Human infections (diagnosis)
Argentina	Endemic	Bovines (serology) [1,2]<br>Dogs (serology) [3]<br>Ticks (PCR) [4]	Q fever, 1 case (serology) [5]<br>Pneumonia, 1 case (serology) [6]
Brazil	Endemic	Bovines (serology) [1]<br>Domestic animals (PCR) [7]	Healthy individuals (serology) [8-11]<br>HIV+ individuals (serology) [12]<br>Pneumonia, 1 case (PCR) [13]<br>Endocarditis, 2 cases (serology) [14,15]<br>Endocarditis, 1 case (PCR) [16]<br>Fever of unknown origin, 1 case (PCR) [17]<br>Outbreak (serology) ([18]
Bolivia	NR (2014)	-	-
Chile	Endemic	-	-
Colombia	Endemic	Bovines (serology) [19]	Healthy individuals (serology) [20,21]<br>Endocarditis, 1 case (serology) [22]<br>Pneumonia,1 case (serology) [23]
Ecuador	NR (2014)	Cow milk (PCR) [24]	Febrile patients (serology) [25]
French Guiana	No information	Three-toed sloth, ticks (PCR) [26]	Pneumonia, endocarditis (serology, PCR) [27][b]
Guyana	NR (2013)	-	-
Paraguay	NR (2014)	-	-
Peru	NR (2014)	-	Healthy individuals (serology) [28]<br>Fever, 1 case (serology) [28]
Suriname	NR (2014)	-	-
Uruguay	Endemic	Bovines, goats, swine (serology) [29]	Various outbreaks (serology) [29]<br>Healthy individuals (serology) [29]<br>Endocarditis, 1 case (serology) [30]<br>Outbreak (serology) [31]
Venezuela	NR (2014)	Goats (serology) [32]	Healthy individuals (serology) [1]<br>Respiratory infections (serology) [33]

NR, Never reported

[a] World Organization for Animal Health (OIE) information on Q fever in animals by country (year)

[b] Various reports on human infections in French Guiana, for review see reference 27

References

1. Kaplan MM, Bertagna P. The geographical distribution of Q fever. Bull World Health Organ 1955;13:829-60.
2. Trezeguet MA, Debenedetti RT, Suarez MF, Barral LE, Ramos M. Detección de fiebre Q en majadas generales caprinas, en la República Argentina. Revista Veterinaria Argentina 2010;27:1-9.
3. Cicuttin GL, Lobo B, Anda P, Jado García I. Seropositividad a *Coxiella burnetii* (agente de la fiebre Q) en caninos domésticos de la Ciudad Autónoma de Buenos Aires. Invet 2013;15:131-6.
4. Pacheco RC, Echaide IE, Alves RN, Beletti ME, Nava S, Labruna MB. *Coxiella burnetii* in ticks, Argentina. Emerg Infect Dis 2013;19:344-6.
5. Romana C, Roldan L, Torrico R, Mayer H. First acute case of Q fever diagnosed in Argentina (preliminary note). Sem Med 1959;115:506-7.
6. Luna CM, Famiglietti A, Absi R, Videla AJ, Nogueira FJ, Fuenzalida AD, Gené RJ. Community-acquired pneumonia: etiology, epidemiology, and outcome at a teaching hospital in Argentina. Chest 2000;118:1344-54.
7. Mares-Guia MA, Rozental T, Guterres A, Gomes R, Almeida DN, Moreira NS, Barreira JD, Favacho AR, Santana AL, Lemos ER. Molecular identification of the agent of Q fever - *Coxiella burnetii* - in domestic animals in State of Rio de Janeiro, Brazil. Rev Soc Bras Med Trop 2014;47:231-4.
8. Ribero-Neto A, Nikitin T, Ribeiro IF. Estudo sobre a febre Q em São Paulo. III. Prevalência em ordenhadores e tratadores de bovinos. Rev Inst Med Trop S Paulo 1964; 6:255-257.
9. Riemann HP, Brant PC, Behymer DE, Franti CE. *Toxoplasma gondii* and *Coxiella burneti* antibodies among Brazilian slaughterhouse employees. Am J Epidemiol 1975;102:386-93.
10. Riemann HP, Brant PC, Franti CE, Reis R, Buchanan AM, Stormont C, Behymer DE. Antibodies to *Toxoplasma gondii* and *Coxiella burneti* among students and other personnel in veterinary colleges in California and Brazil. Am J Epidemiol 1974;100:197-208.
11. Da Costa PS, Brigatte ME, Greco DB. Antibodies to Rickettsia rickettsii, Rickettsia typhi, *Coxiella burnetii, Bartonella henselae, Bartonella quintana*, and *Ehrlichia chaffeensis* among healthy population in Minas Gerais, Brazil. Mem Inst Oswaldo Cruz 2005;100:853-9.
12. Lamas CC, Rozental T, Bóia MN, Favacho AR, Kirsten AH, da Silva AP, de Lemos ER. Seroprevalence of *Coxiella burnetii* antibodies in human immunodeficiency virus-positive patients in Jacarepaguá, Rio de Janeiro, Brazil. Clin Microbiol Infect 2009;15 (Suppl 2):140-1.
13. Rozental T, Mascarenhas LF, Rozenbaum R, Gomes R, Mattos GS, Magno CC, Almeida DN, Rossi MI, Favacho AR, de Lemos ER. *Coxiella burnetii*, the agent of Q fever in Brazil: its hidden role in seronegative arthritis and the importance of molecular diagnosis based on the repetitive element IS1111 associated with the transposase gene. Mem Inst Oswaldo Cruz 2012;107:695-7.
14. Siciliano RF, Ribeiro HB, Furtado RH, Castelli JB, Sampaio RO, Santos FC, Colombo S, Grinberg M, Strabelli TM. Endocarditis due to *Coxiella burnetii* (Q fever): a rare or underdiagnosed disease? Case report. Rev Soc Bras Med Trop 2008;41:409-12.
15. Siciliano RF, Strabelli TM, Zeigler R, Rodrigues C, Castelli JB, Grinberg M, Colombo S, da Silva LJ, Mendes do Nascimento EM, Pereira dos Santos FC, Uip DE. Infective endocarditis due to *Bartonella* spp. and *Coxiella burnetii*: experience at a cardiology hospital in Sao Paulo, Brazil. Ann N Y Acad Sci 2006;1078:215-22.
16. Lamas Cda C, Ramos RG, Lopes GQ, Santos MS, Golebiovski WF, Weksler C, Ferraiuoli GI, Fournier PE, Lepidi H, Raoult D. *Bartonella* and *Coxiella* infective endocarditis in Brazil: molecular evidence from excised valves from a cardiac surgery referral center in Rio de Janeiro, Brazil, 1998 to 2009. Int J Infect Dis 2013;17:e65-6.
17. Lemos ER, Rozental T, Mares-Guia MA, Almeida DN, Moreira N, Silva RG, Barreira JD, Lamas CC, Favacho AR, Damasco PV. Q fever as a cause of fever of unknown origin and

thrombocytosis: first molecular evidence of *Coxiella burnetii* in Brazil. Vector Borne Zoonotic Dis 2011;11:85-7.
18. da Costa PS, Brigatte ME, Greco DB. Questing one Brazilian query: reporting 16 cases of Q fever from Minas Gerais, Brazil. Rev Inst Med Trop Sao Paulo 2006;48:5-9.
19. de Ruiz HL. Q fever in Colombia, S.A. A serological survey of human and bovine populations. Zentralbl Veterinarmed B 1977;24:287-92.
20. Máttar S, Parra M. Detection of antibodies to *Anaplasma*, *Bartonella* and *Coxiella* in rural inhabitants of the Caribbean area of Colombia. Rev MVZ Cordoba 2006;11:781-9.
21. Mattar S, Contreras V, González M, Camargo F, lvarez JA, Oteo JA. Infection by *Coxiella burnetii* in a patient from a rural area of Monteria, Colombia. Rev Salud Pública 2014;16:789-92.
22. Betancur CA, Múnera AG. *Coxiella burnetii* endocarditis: Q fever. Act Med Colomb 2012;37:31-3.
23. Meza-Cardona JC, Rosso-Suárez F. Neumonía por *Coxiella burnetii*: presentación de un caso y revisión de la literatura. Rev CES Med 2012;26:201-7.
24. Rojas MI, Barragán V, Trueba G, Hornstra H, Pearson T, Keim P. Detección de *Coxiella burnetii* en leche de bovinos domésticos del Ecuador. Avances 2013,5:B5-B9.
25. Manock SR, Jacobsen KH, de Bravo NB, Russell KL, Negrete M, Olson JG, Sanchez JL, Blair PJ, Smalligan RD, Quist BK, Espín JF, Espinoza WR, MacCormick F, Fleming LC, Kochel T. Etiology of acute undifferentiated febrile illness in the Amazon basin of Ecuador. Am J Trop Med Hyg 2009;81:146-51.
26. Davoust B, Marié JL, Pommier de Santi V, Berenger JM, Edouard S, Raoult D. Three-toed sloth as putative reservoir of *Coxiella burnetii*, cayenne, French Guiana. Emerg Infect Dis 2014;20:1760-1.
27. Eldin C, Mahamat A, Demar M, Abboud P, Djossou F, Raoult D. Q fever in French Guiana. Am J Trop Med Hyg 2014;91:771-6.
28. Blair PJ, Schoeler GB, Moron C, Anaya E, Caceda R, Cespedes M, Cruz C, Felices V, Guevara C, Huaman A, Luckett R, Mendoza L, Richards AL, Rios Z, Sumner JW, Villaseca P, Olson JG. Evidence of rickettsial and leptospira infections in Andean northern Peru. Am J Trop Med Hyg 2004;70:357-63.
29. Somma-Moreira RE, Caffarena RM, Somma S, Pérez G, Monteiro M. Analysis of Q fever in Uruguay. Rev Infect Dis 1987;9:386-7.
30. Moreira Eglinger LE, Braselli A. Fiebre Q - Endocarditis a *Coxiella burnetii* - Primera comunicación nacional. Rev Med Uruguay 1994;10:131-7.
31. Hernández S, Lyford-Pike V, Alvarez ME, Tomasina F. Q fever outbreak in an experimental wildlife breeding station in Uruguay. Revista de Patologia Tropical 2007;36:129-40.
32. Oropeza M, Dickson L, Maldonado J, Kowalski A. Seropositividad a *Coxiella burnetii* en cabras de la parroquia Trinidad Samuel del municipio Torres, estado Lara, Venezuela. Zootecnia Trop 2010;28:557-60.
33. Gotera Zambrano JL, Mavárez Montes AR, Bermúdez Fereira JE, Durán Mojica AA, Valero Cedeño NJ. Seroprevalencia de virus respiratorios y bacterias atípicas en una población del estado Zulia, Venezuela. Revista de la Sociedad Venezolana de Microbiología 2012;32:148-52.
